# Epithelial Heparan Sulfate Promotes *Staphylococcus aureus* Corneal Infection by Inhibiting Cathelicidins

**DOI:** 10.1002/pgr2.70041

**Published:** 2025-11-03

**Authors:** Kazutaka Hayashida, Jeffrey D. Esko, Richard D. Gallo, Jian Liu, Winston W.-Y. Kao, Pyong Woo Park

**Affiliations:** 1 Department of Medicine, Boston Children’s Hospital, Boston, Massachusetts, USA; 2 Department of Cellular and Molecular Medicine, University of California San Diego, La Jolla, California, USA; 3 Department of Dermatology, University of California San Diego, La Jolla, California, USA; 4 Division of Chemical Biology and Medicinal Chemistry, University of North Carolina Chapel Hill, Chapel Hill, North Carolina, USA; 5 Department of Ophthalmology, University of Cincinnati, Cincinnati, Ohio, USA; 6 Department of Pediatrics, Harvard Medical School, Boston, Massachusetts, USA

**Keywords:** antimicrobial peptide, cathelicidin, glycosaminoglycan, heparan sulfate, host defense, *Staphylococcus aureus*

## Abstract

Cathelicidins are short cationic peptides with potent microbicidal activities and comprise an important arm of host innate immunity. Many cell types can produce cathelicidins, but they are mainly expressed by recruited immune cells and are induced in epithelial cells during infection. Although the mechanisms of bacterial killing by cathelicidins have been largely elucidated in vitro, those that regulate their activities in vivo are less well understood. Bacterial pathogens often co-opt host extracellular matrix (ECM) components and their functions to escape host defense; however, it is unclear whether such mechanisms exist against cathelicidins. Several studies have demonstrated that host heparan sulfate (HS) inhibits LL-37, the human cathelicidin, suggesting that bacteria might exploit HS to evade killing by cathelicidins. However, precisely how HS inhibits LL-37 and possibly other cathelicidins remains unknown, and the role of the HS-cathelicidin interaction in infectious disease has not been rigorously studied. Here, we found that deleting CRAMP, the murine cathelicidin, significantly increases the susceptibility of mice to *Staphylococcus aureus* corneal infection. We also determined that heparan compounds bind to CRAMP with low nanomolar affinity, the secondary structure of CRAMP is required for HS binding, and HS binding to CRAMP inhibits CRAMP binding to target bacterial cells. Furthermore, we found that heparan compounds inhibit the killing of *S. aureus* by cathelicidins derived from several mammalian species in a 2-*O*-sulfate-dependent manner. Additionally, we demonstrate for the first time that conditional deletion of HS2ST, the enzyme responsible for 2-*O*-sulfation of HS, in corneal epithelial cells significantly reduces the susceptibility of mice to corneal infection. Altogether, these data uncover an endogenous inhibition mechanism of cathelicidins where 2-*O*-sulfated epithelial HS tightly binds and neutralizes the antibacterial activity of cathelicidins.

## 1 | Introduction

The mucosal epithelium serves as a vital interface where the host comes into direct contact with potential environmental hazards, including microbial pathogens. At this juncture, the physical barrier created by mucus, glycocalyx, and cellular junctions, along with the innate immune system, forms the first line of defense against microbial threats. One of the key components of innate immunity is the antimicrobial peptides (AMPs) [1], which are also called host defense peptides (HDPs) [2] and cationic host defense peptides (CHDPs) [3]. AMPs in their mature form are typically amphipathic small peptides with fewer than 50 amino acids and a net positive charge of +2 or higher at physiological pH. AMPs are produced by most species, including animals and plants, and play important roles in the host defense against pathogens, including viruses, bacteria, and fungi [1, 3–5]. AMPs also coordinate inflammatory responses to infection and tissue injury [3, 5–7], suggesting that they defend against infections both directly via their antimicrobial activity and indirectly via their pro-inflammatory activity.

One of the most prominent AMPs is the cathelicidins, named after the conserved N-terminal cathelin domain, which was first purified as a cathepsin L inhibitor. Cathelicidins are produced as prepropeptides containing an N-terminal signal peptide, a cathelin-like domain, and the C-terminal AMP. The pro-cathelin-like domain is enzymatically cleaved off once the peptide is secreted to generate the mature cathelicidin AMP [8]. Cathelicidins are expressed by many cell types, including neutrophils, mast cells, dendritic cells, epithelial cells, fibroblasts, neurons, astrocytes, microglia, meningeal cells, and adipocytes [5, 9–12], reflecting their key functions in infection and inflammation in various tissues. However, during most infections, cathelicidins are expressed primarily by infected epithelial cells and resident and recruited immune cells.

Preclinical studies using mice deficient in CRAMP, the murine cathelicidin, have demonstrated the critical role of cathelicidins in controlling infections. For example, CRAMP-deficient mice are hypersusceptible to: necrotizing skin infection caused by *Streptococcus pyogenes* [13]; keratitis [14] and pneumonia [15] caused by *Pseudomonas aeruginosa*; pneumonia caused by *Klebsiella pneumoniae* [16]; urinary tract infection caused by *E. coli* [17]; intestinal tract infection caused by *Citrobacter rodentium* [18]; mastitis caused by *Staphylococcus aureus* [19]; meningitis caused by *Streptococcus pneumoniae* [20]; and viral lung [21] and skin [22] infection by RSV and vaccinia virus, respectively. Unlike antibiotics, the chances of bacterial pathogens developing resistance to AMPs are believed to be low due to their rather broad antibacterial mechanisms, which target bacterial membranes through electrostatic interactions, followed by membrane disruption. These features of AMPs recommend them as potential antibacterial drugs.

However, pathogens are known to find ways to overcome host defense mechanisms, including AMPs, for their survival. A common mechanism used for AMP resistance is the incorporation of positively charged modifications into cell surface components, which reduces the affinity for cationic AMPs. For example, *S. aureus* uses the *dlt* operon to D-alanylate cell wall teichoic acids [23] and MprF to enzymatically modify membrane phospholipids with Lys residues [24]. *S. aureus* can also proteolytically degrade cathelicidins [25]. Furthermore, we previously found that *S. aureus* induces the ectodomain shedding of syndecan-1, a major heparin sulfate proteoglycan (HSPG) of epithelial cells [26], and syndecan-1 ectodomains inhibit CRAMP in a heparan sulfate (HS)-dependent manner [27]. We also found that mice deficient in syndecan-1 are significantly less susceptible to *S. aureus* corneal infection [27, 28]. HS also inhibits the antibacterial activity of the human cathelicidin, LL-37, in human wound fluids [29], septic plasma [30], and bronchoalveolar lavage fluids from cystic fibrosis patients [31]. Consistent with these findings, bacterial pneumonia-induced shedding of epithelial HS was found to inhibit the bactericidal activity of cathelicidin [32]. However, how HS inhibits cathelicidins is not understood. Additionally, the role of CRAMP in *S. aureus* corneal infection has not been determined, and the importance of HS inhibition of cathelicidins in vivo remains to be directly tested.

All HS in vivo is found covalently conjugated to HSPG core proteins [33]. HSPGs are expressed on the cell surface, in intracellular compartments, and in the extracellular matrix (ECM) [33]. HSPGs at the cell surface function primarily as coreceptors for HS-binding molecules, whereas HSPG ectodomains shed from the surface regulate HS-protein interactions in an autocrine or paracrine manner [34–36]. In HS biosynthesis, a non-sulfated HS precursor is polymerized on specific Ser residues of HSPG core proteins and then extensively and variably modified by *N*-deacetylases, epimerase, and *N*- and *O*-sulfotransferases [37], resulting in highly heterogeneous, mature HS chains. The modifications of HS are thought to enable HSPGs to interact specifically with many molecules. Here, we identify a key modification of HS for cathelicidin binding and inhibition, and show that corneal epithelial HS promotes *S. aureus* pathogenesis in the cornea by inhibiting CRAMP.

## 2 | Results

### 2.1 | Mice Deficient in CRAMP Are Hypersusceptible to *S. aureus* Corneal Infection

**FIGURE 1 |:**
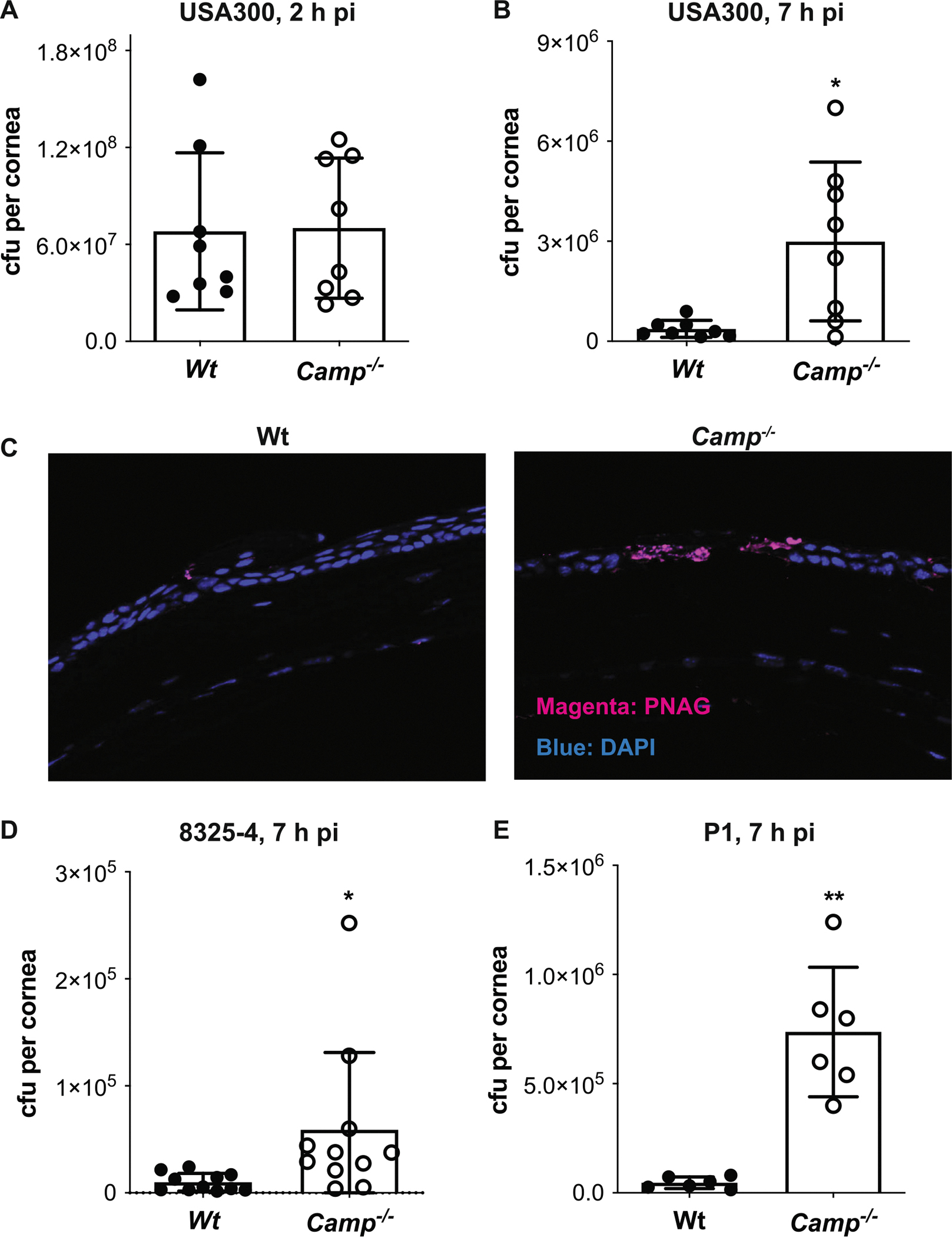
*Camp*^−/−^ mice are hypersusceptible to *Staphylococcus aureus* corneal infection. Injured corneas of Wt and *Camp*^−/−^ mice were infected topically with *S. aureus* strains. Corneal infection with 3 × 10^7^ cfu of *S. aureus* USA300 was assessed by corneal cfu counts at (A) 2 h pi or (B) 7 h pi (*n* = 8, *p < 0.05, Welch’s *t*-test) and (C) by immunostaining infected corneal sections (7 h pi) with anti-PNAG antibodies to visualize *S. aureus* (magenta = PNAG, blue = DAPI, ×200 original magnification). Note that *S. aureus* primarily infects injured areas in the corneal epithelium of *Camp*^−/−^ mice. The corneal bacterial burden in mice infected with 3 × 10^7^ cfu of *S. aureus* (D) 8325–4 or (E) P1 was determined at 7 h pi (*n* = 6–9, *p < 0.05, **p < 0.01, Welch’s *t*-test).

Available data suggest that AMPs are key host determinants that protect against infectious keratitis at the ocular surface [38], and cathelicidins kill *S. aureus* in vitro [39]. We first examined whether CRAMP is indeed critical in the clearance of *S. aureus* from infected corneas in a mouse model of injury-induced corneal infection. Corneas of Wt and CRAMP null (*Camp*^−/−^) mice were injured by one vertical scratch with a 29 G needle and then infected topically with 3 × 10^7^ cfu of methicillin-resistant *S. aureus* (MRSA) USA300. At various times postinfection (pi), the corneal bacterial burden was measured. The bacterial burden was similar at 2 h pi between Wt and *Camp*^−/−^ corneas (Figure 1A), suggesting that CRAMP does not affect the initial colonization of bacteria to injured corneas. However, the corneal bacterial load was significantly higher by eightfold in *Camp*^−/−^ mice compared to Wt mice at 7 h pi (Figure 1A), indicating that CRAMP is required for the rapid clearance of bacteria. Immunostaining with an antibody directed against poly-β-1,6-*N*-acetylglucosamine (PNAG), a cell surface polysaccharide expressed by many bacterial pathogens including *S. aureus* [40, 41] but not by host cells, showed intense staining for *S. aureus* at the site of epithelial injury confirming the elevated cfu counts in *Camp*^−/−^ corneas compared to Wt corneas (Figure 1B). *Camp* expression was significantly increased in Wt corneas at 4 h pi compared to uninfected Wt corneas (Figure S1A). *Camp*^−/−^ corneas were also hypersusceptible to infection by a laboratory strain (8325–4) (Figure 1D) and a clinical blood isolate (P1) (Figure 1E) of *S. aureus* by 6- and 16-fold, respectively, when compared to Wt corneas, demonstrating that the ability of CRAMP to protect against *S. aureus* corneal infection is not strain-dependent. Together, these results suggest that CRAMP-mediated bacterial killing is a crucial host defense mechanism against *S. aureus* corneal infection.

### 2.2 | Survey of Anti-Staphylococcal Cathelicidins

Next, we examined the dose response to cathelicidins from different species, which can have relatively large differences in primary sequence. However, in general, cathelicidins are similarly cationic, α-helical, and amphipathic, and have similar microbial killing profiles [39, 42, 43]. For example, mature mouse CRAMP only has 48% homology with mature human LL-37, but they have similar amphipathic α-helical structures and antimicrobial spectra. We tested the effects of HS on *S. aureus* killing by CRAMP (pI: 10.0), LL-37 (pI: 10.5) [44], rabbit CAP-18 (pI: 11.9) [39], and sheep SMAP-29 (pI: 12.7) [42]. Greater than 98% of *S. aureus* were killed when incubated with 0.5 μM of CRAMP, CAP-18, or SMAP-29 and 1 μM of LL-37 for 2 h at 37°C (Figure S1B). These effective doses are consistent with the concentration of cathelicidins found in biological fluids. For instance, the concentration of LL-37 in tracheal aspirates of newborns [45] and seminal plasma [46] is 20 μg/mL (4.4 μM) and 85 μg/mL (18.9 μM), respectively, and the activity of LL-37 against bacterial pathogens is generally in the submicromolar to micromolar range [47].

### 2.3 | 2-O-Sulfate Motifs in HS/HP Inhibit the Anti-Staphylococcal Activity of Cathelicidins

**FIGURE 2 |:**
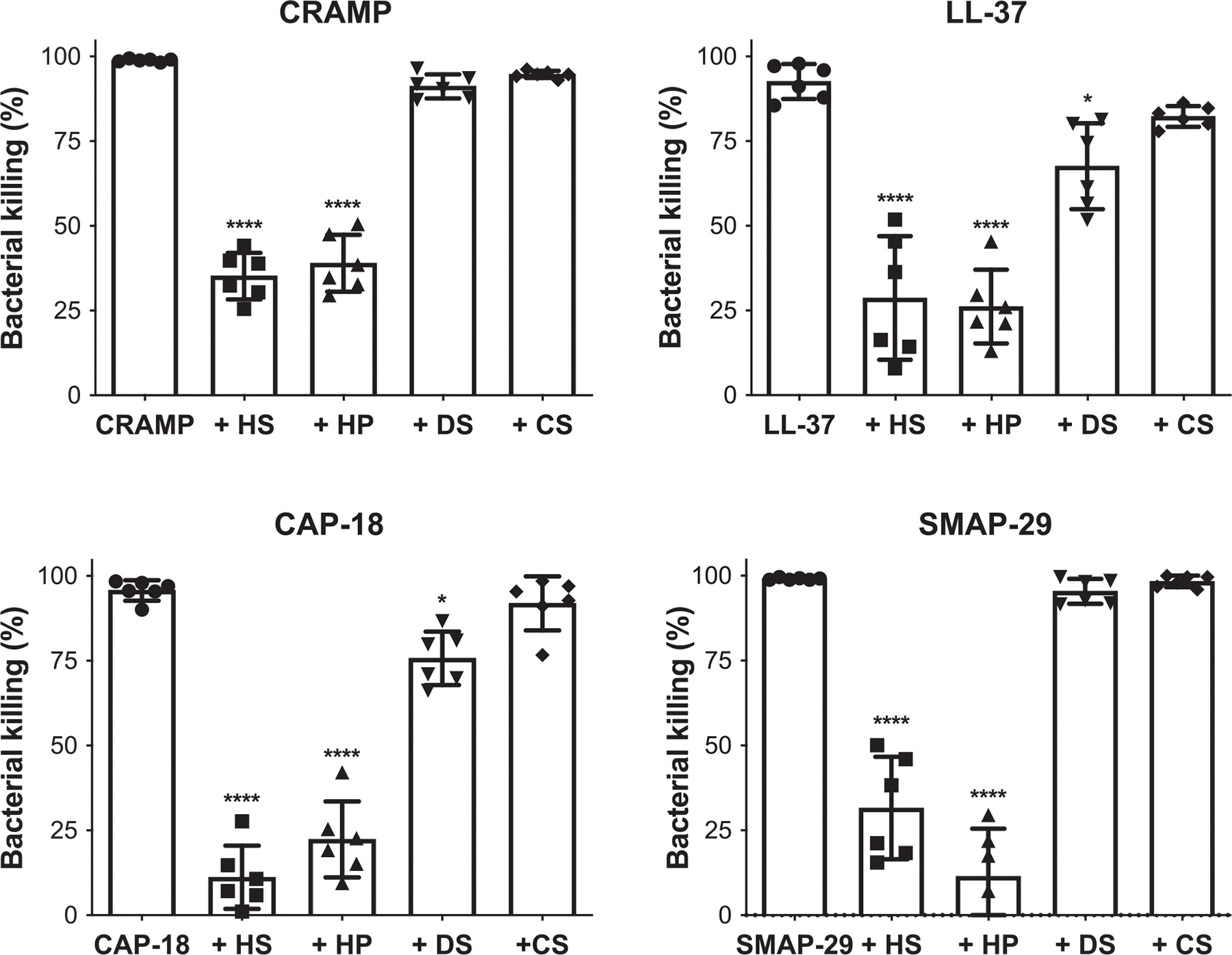
HS and HP significantly inhibit *Staphylococcus aureus* killing by cathelicidins from different species. Late-log phase *S. aureus* USA300 (~500 cfu) was incubated with 0.5 μM of CRAMP, LL-37, or SMAP-29 or 1 μM LL-37 without or with 5 μg/mL of HS, HP, DS, or CS in phosphate buffer (8.2 mM Na_2_HPO_4_, 1.8 mM KH_2_PO_4_, pH 7.4) for 2 h at 37°C. Samples were plated onto TSA plates, and viable bacteria (cfu) were counted the following day. Data are shown as % bacterial killing, where killing activity in the presence of cathelicidin only represents 100% (*n* = 6, *p < 0.05, ****p < 0.0001, one-way ANOVA, multiple comparisons).

Titration studies showed that porcine mucosal HS inhibits *S. aureus* killing by CRAMP in a dose-dependent manner, where 5 μg/mL (~0.5 μM) HS significantly inhibited bacterial killing by 0.5 μM CRAMP (Figure S2A). Based on these results, we tested the effects of 5 μg/mL of HS, heparin (HP), dermatan sulfate (DS), and chondroitin sulfate A (CS) on *S. aureus* killing by cathelicidins. Both HS and HP significantly and similarly inhibited *S. aureus* killing by CRAMP, LL-37, CAP-18, and SMAP-29 (Figure 2). DS did not inhibit *S. aureus* killing by CRAMP and SMAP-29, and CS had no significant effect on any of the four cathelicidins at the doses tested (Figure 2). DS significantly inhibited *S. aureus* killing by LL-37 and CAP-18 by 27% and 21%, respectively. Still, these effects were much less pronounced compared to the over 70% inhibition observed with HS or HP (Figure 2). HS and HP did not inhibit bacterial killing by cathelicidins when pre-incubated with bacteria and washed away before incubation with the cathelicidins (Figure S2B), establishing that the inhibitory effects of HS and HP are on the peptides and not on bacteria. Together, these results suggest that HS/HP directly and potently inhibit the staphylocidal activity of cathelicidins.

**FIGURE 3 |:**
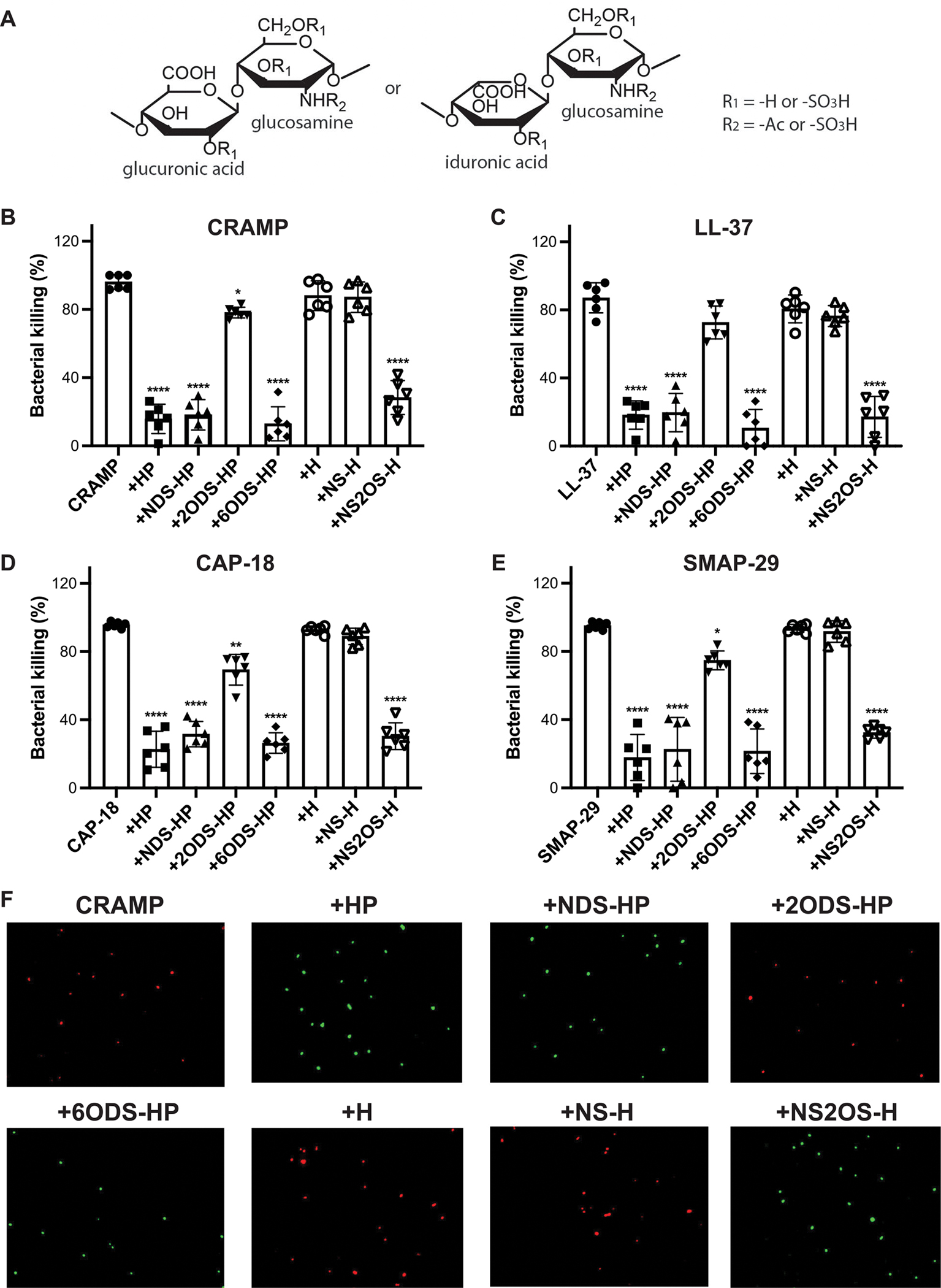
Heparan compounds inhibit cathelicidins primarily through their 2-*O*-sulfated motifs. (A) Schematics of HS/HP structure. Late-log phase *Staphylococcus aureus* USA300 (~500 cfu) was incubated with (B) 0.5 μM CRAMP, (C) 1 μM LL-37, (D) 0.5 μM CAP-18, or (E) 0.5 μM SMAP-29 without or with 5 μg/mL HP, NDS-HP, 2ODS-HP, 6ODS-HP, H, NS-H, or NS2OS-H for 2 h at 37°C and bacterial killing was measured by counting viable bacteria plated on TSA plates the following day (*n* = 6, *p < 0.05, **p < 0.01, ****p < 0.0001, one-way ANOVA, multiple comparisons). (F) BaclightGreen-labeled *S. aureus* (~400 cfu) was incubated with 0.5 μM CRAMP without or with 5 μg/mL HP, NDS-HP, 2ODS-HP, 6ODS-HP, H, NS-H, or NS2OS-H for 30 min at room temperature. Bacteria were washed, incubated with 10 μg/mL propidium iodide for 15 min at 4°C, and live (green) and dead (red) bacteria were visualized by fluorescence microscopy.

Next, we pursued the structural features of HS/HP that are essential for inhibiting cathelicidins by testing the effects of modified HP. The structure of HS/HP is shown in Figure 3A. Chemical removal of *N*- or 6-*O*-sulfates did not alter the ability of HP to significantly inhibit *S. aureus* killing by CRAMP, LL-37, CAP-18, and SMAP-29 (Figure 3B–E). However, 2-*O*-desulfation significantly inhibited the ability of HP to inhibit *S. aureus* killing by all cathelicidins tested (Figure 3B–E). To investigate the importance of 2-*O*-sulfation further, we tested the effects of chemoenzymatically sulfated heparosan (H) compounds. H is a polysaccharide isolated from *E. coli* that is identical in structure to nonsulfated HS. H and *N*-sulfated H showed minimal effect on bacterial killing by the four cathelicidins. On the other hand, *N*- and 2-*O*-sulfated H significantly inhibited *S. aureus* killing by all cathelicidins tested (Figure 3B–E). Bacterial killing by CRAMP and inhibition of CRAMP killing by HP and H compounds were also visualized by live/dead staining using Baclight green and propidium iodide (red). Most bacteria incubated with CRAMP only or CRAMP with HP and H compounds lacking 2-*O*-sulfates were dead (red), whereas those incubated with CRAMP and HP and H compounds containing 2-*O*-sulfates were alive (green) (Figure 3F), confirming the cfu data. Individual images for the live/dead staining are shown in Figure S3. Because removal of *N*-sulfates from HP and addition of *N*-sulfates to H did not affect their ability to alter *S. aureus* killing by cathelicidins, these data suggest that the inhibitory activity of HS is not solely dependent on the overall sulfation of HS and HP. More importantly, these data indicate that HS selectively inhibits cathelicidins via its 2-*O*-sulfated motifs.

### 2.4 | HP Binds to CRAMP and Interferes With CRAMP Binding to *S. aureus*

**FIGURE 4 |:**
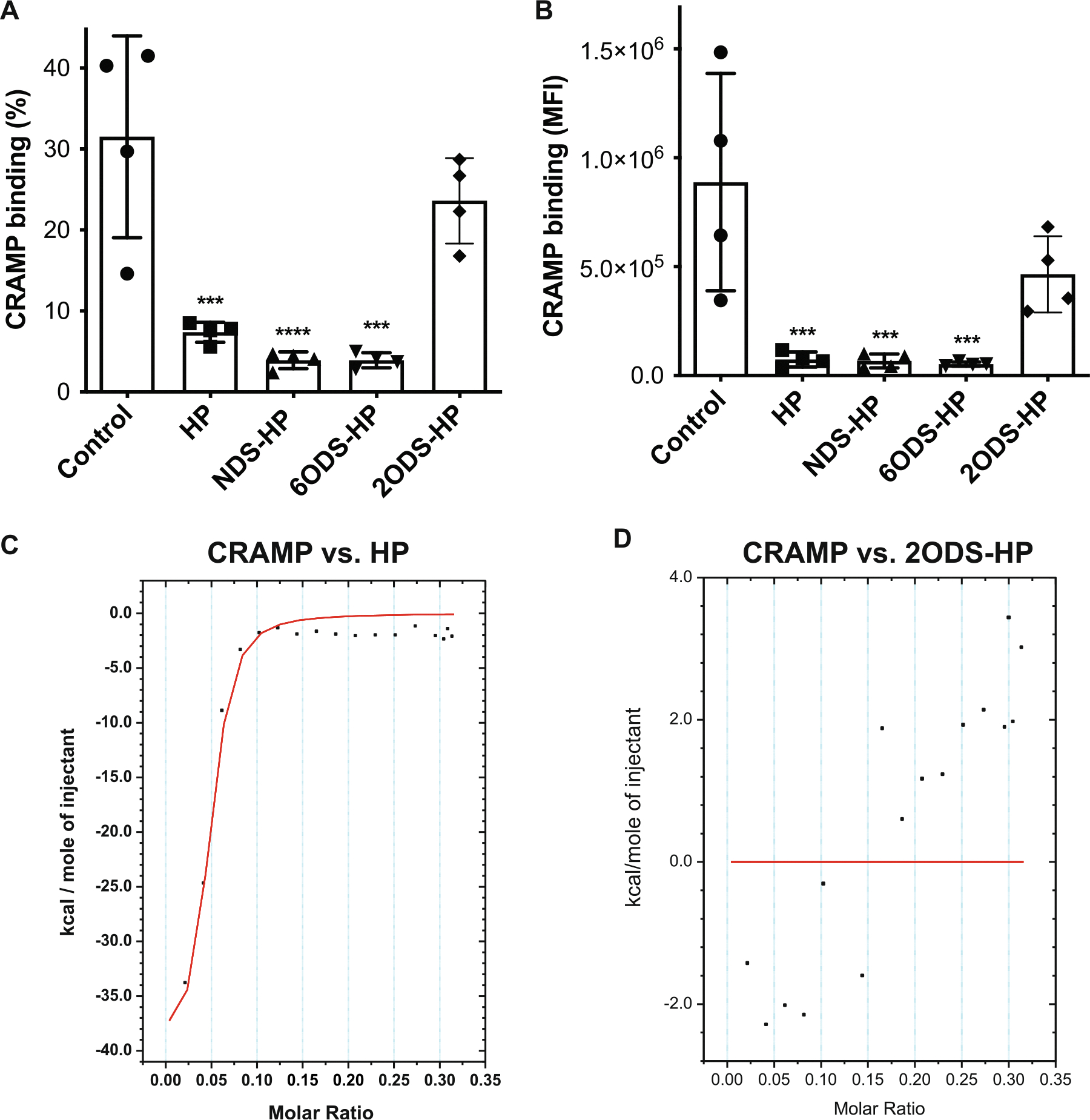
2-*O*-sulfated heparan compounds directly bind to CRAMP with high affinity. Alexa 647-conjugated CRAMP (0.5 μM) was incubated with Affi-Gel HP beads without or with 20 μg/mL of HP, NDS-HP, 2ODS-HP, or 6ODS-HP for 1 h at room temperature. CRAMP fluorescence associated with Affi-Gel HP beads was analyzed by flow cytometry. Results are shown as (A) % CRAMP binding and (B) mean fluorescence intensity (MFI) of CRAMP binding (*n* = 4, ***p < 0.001, ****p < 0.0001). ITC analyses for CRAMP binding to HP were performed at 25°C using a MicroCal iTC 200 titration calorimetry. The fitted heat curves of titrating CRAMP binding to (C) HP and (D) 2ODS-HP are shown.

We next examined if HS binds directly to CRAMP and if this interaction is dependent on select sulfation motifs. Alexa 647-conjugated CRAMP was incubated with HP affinity beads without or with excess HP, *N*-desulfated HP (NDS-HP), 2-*O*-desulfated HP (2ODS-HP), or 6-*O*-desulfated HP (6ODS-HP) for 1 h, and CRAMP binding was measured by flow cytometry. CRAMP bound avidly to HP beads (31.5% of input), but this was significantly inhibited by HP, NDS-HP, and 6ODS-HP by over 75%, whereas 2ODS-HP only reduced binding by 25% (Figure 4A). The mean fluorescence intensity (MFI) was similarly reduced by HP compounds containing 2-*O*-sulfates by over 91% compared to control, but not by 2ODS-HP (Figure 4B), indicating that HP binds directly to CRAMP in a 2-*O*-sulfate-dependent manner.

Next, we assessed thermodynamic parameters of the HP-CRAMP interaction by isothermal titration calorimetry (ITC). CRAMP was titrated with HP and the fitted heat curve resulting from the interaction revealed that binding is saturable and with an enthalpy change of −40 kcal/mol, binding stoichiometry of 25 moles of CRAMP per mole of HP, and a binding affinity of 45.2 nM for CRAMP binding to HP (Figure 4C), which is in the high affinity range compared to other reported HS/HP–protein interactions [48]. Incubation of CRAMP with 2ODS-HP did not produce significant heat changes (Figure 4D), providing further evidence that HP and HS bind to CRAMP through their 2-*O*-sulfated motifs.

**FIGURE 5 |:**
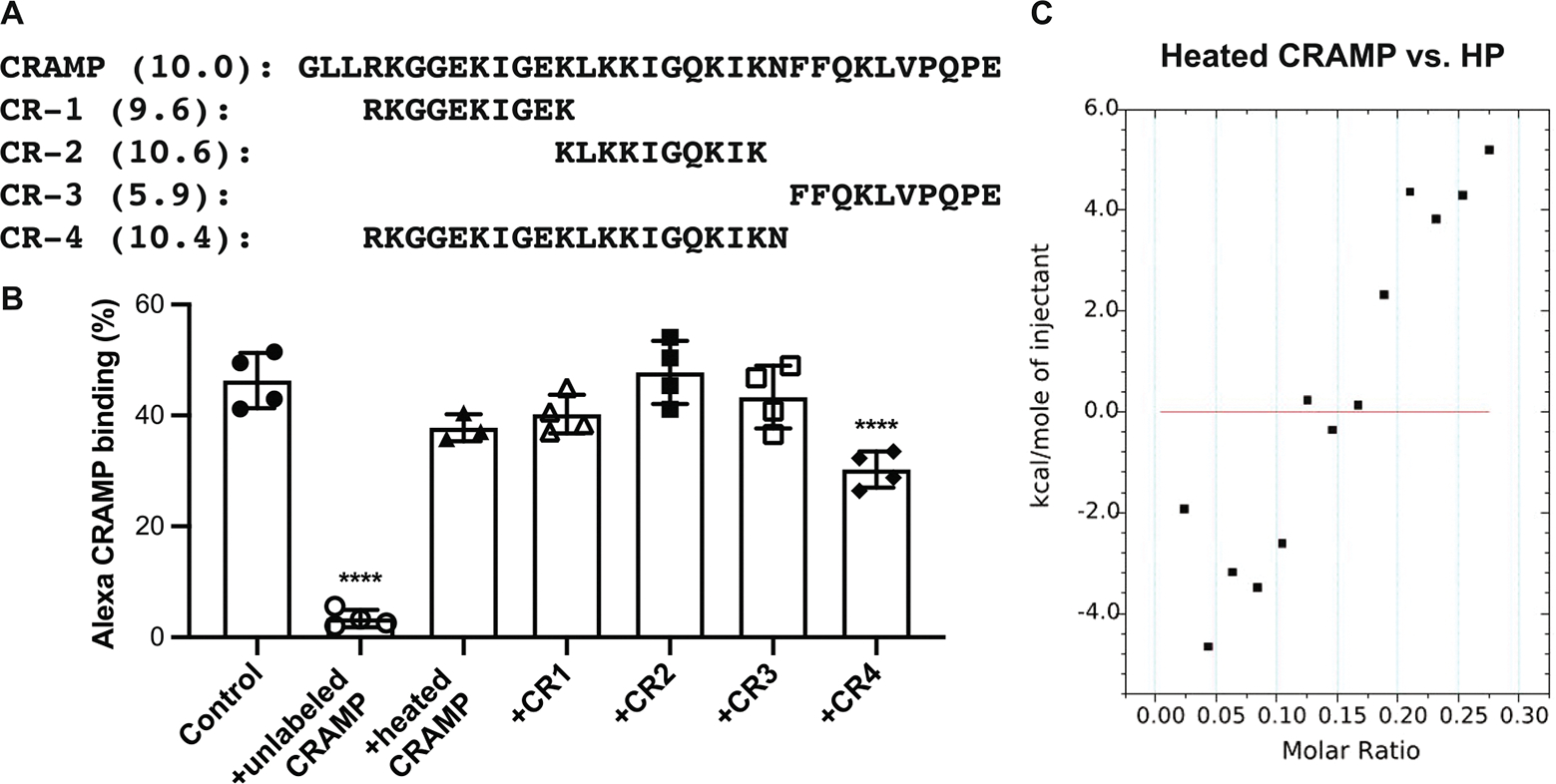
Secondary structure of mature CRAMP is required for binding to heparan compounds. (A) Sequence and pI (in parentheses) of the 34 amino acid long mature CRAMP and four 10- or 20-mer peptides that span amino acids 4–13 (CR-1), 13–22 (CR-2), 24–33 (CR-3), or 4–23 (CR-4) of mature CRAMP. (B) Affi-Gel HP beads were incubated with 250 nM of Alexa 647-labeled CRAMP without or with 5 μM of unlabeled CRAMP, heat-treated CRAMP, CR-1, CR-2, CR-3, or CR-4 peptides for 1 h at room temperature. Results shown are % CRAMP binding to HP affinity beads (*n* = 3–4, ****p < 0.0001, one-way ANOVA, multiple comparisons). (C) ITC analyses of heat-treated CRAMP binding to HP. The fitted heat curve of titrating CRAMP binding to HP is shown.

The finding that a select sulfate modification in HS/HP is required for efficient CRAMP binding suggested that a specific region or structure in CRAMP might mediate its interaction with HS/HP. We pursued this possibility by first testing the ability of truncated CRAMP peptides to inhibit CRAMP binding to HP. We synthesized 10-mer and 20-mer peptides that correspond to amino acids 4–13 (CR-1), 13–22 (CR-2), 24–33 (CR-3), and 4–23 (CR-4) of mature CRAMP (Figure 5A). In the experiment shown, approximately 41% of Alexa 647-labeled, mature CRAMP bound to Affi-Gel HP beads (Figure 5B). Addition of 20-fold excess unlabeled CRAMP and CR4 peptide significantly inhibited Alexa CRAMP binding to HP beads by 92.7% and 34.7%, respectively, but addition of CR-1, CR-2, and CR-3 peptides did not (Figure 5B). Because intact CRAMP (pI: 10.0), CR-1 peptide (pI: 9.6), CR-2 peptide (pI: 10.6), and CR-4 peptide (pI: 10.4) are similarly cationic, these findings indicate that positive charge alone does not drive CRAMP’s interaction with HS/HP. Cathelicidins adopt an amphipathic α-helical structure in physiological buffers, and the extent of α-helicity directly correlates with their antibacterial activity [49]. We therefore tested if disrupting the secondary structure by heating intact CRAMP at 100°C for 20 min would abolish its HP binding activity. Heat-treated CRAMP lost its capacity to inhibit binding of Alexa CRAMP to HP Affigel beads (Figure 5B) and did not show measurable binding to HP by ITC (Figure 5C). These findings indicate that efficient binding of CRAMP to HP requires the full amphipathic α-helix structure, and suggest that shorter CRAMP peptides with some degree of amphipathic α-helicity (CR-4) can also bind to HP, albeit with a lower avidity.

**FIGURE 6 |:**
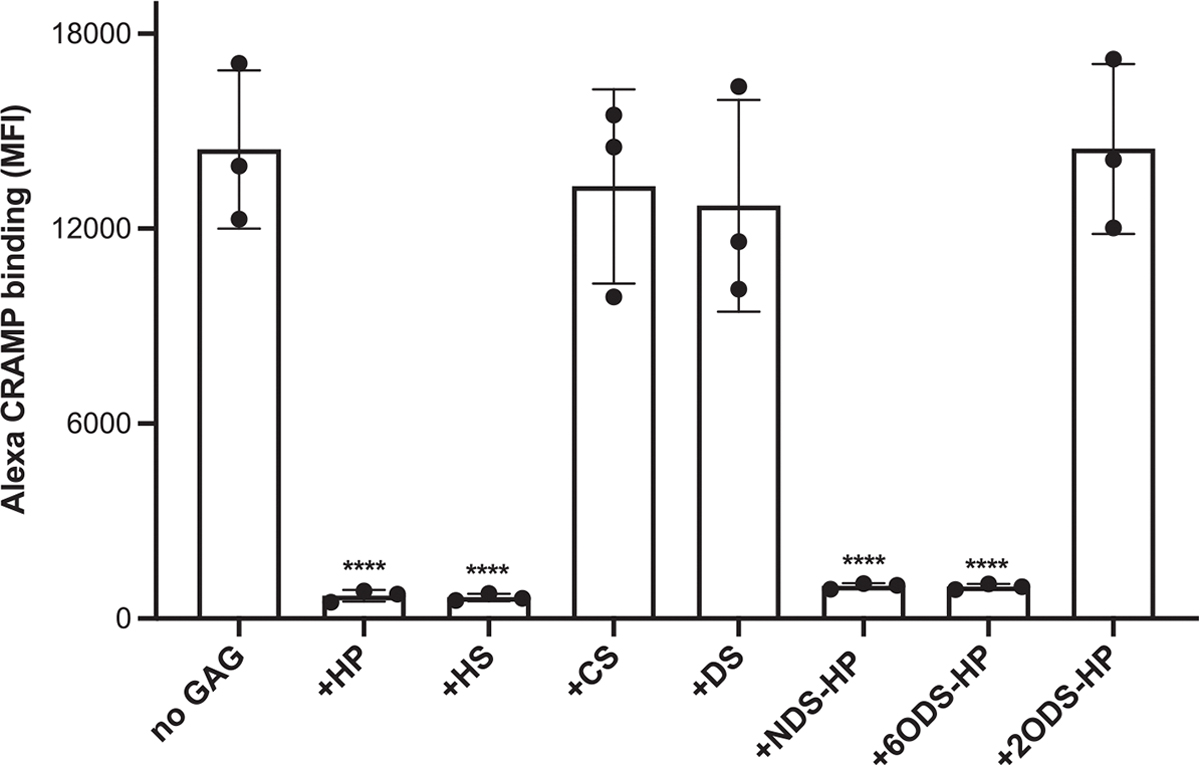
2-*O*-sulfated heparan compounds inhibit CRAMP by interfering with its binding to bacteria. Alexa 647-conjugated CRAMP (0.5 μM) was incubated with 2% paraformaldehyde-fixed *Staphylococcus aureus* USA300 without or with 5 μg/mL HP, HS, CS, DS, NDS-HP, 6ODS-HP, or 2ODS-HP for 1 h at room temperature. *S. aureus*-associated CRAMP fluorescence was analyzed by flow cytometry. Results are shown as % Alexa CRAMP binding (*n* = 3, ****p < 0.0001).

To determine if HS/HP inhibits CRAMP by binding to the AMP and interfering with its ability to bind target bacteria, we next measured binding of Alexa 647-labeled CRAMP to fixed *S. aureus* in the absence or presence of HP, HS, CS, DS, or chemically desulfated HP. Similar to the ability of GAGs to inhibit bacterial killing by CRAMP, heparan compounds containing 2-*O*-sulfates significantly decreased the MFI of Alexa-labeled CRAMP binding to *S. aureus* by over 93% compared to control, whereas 2-*O*-desulfated HP, DS, and CS had no effect (Figure 6A). The proportion of CRAMP-bound bacteria was also markedly reduced by heparan compounds containing 2-*O*-sulfates but not by 2-*O*-desulfated HP, CS, or DS (Figure S4). Together, these results reveal a new pathogenic mechanism in which 2-*O*-sulfated HS motifs bind to CRAMP with high affinity, inhibiting CRAMP binding to *S. aureus* and thereby reducing bacterial killing, which in turn increases *S. aureus* virulence in injured corneas.

### 2.5 | Mice Lacking 2-O-Sulfated HS Motifs Are Significantly Less Susceptible to *S. aureus* Corneal Infection

**FIGURE 7 |:**
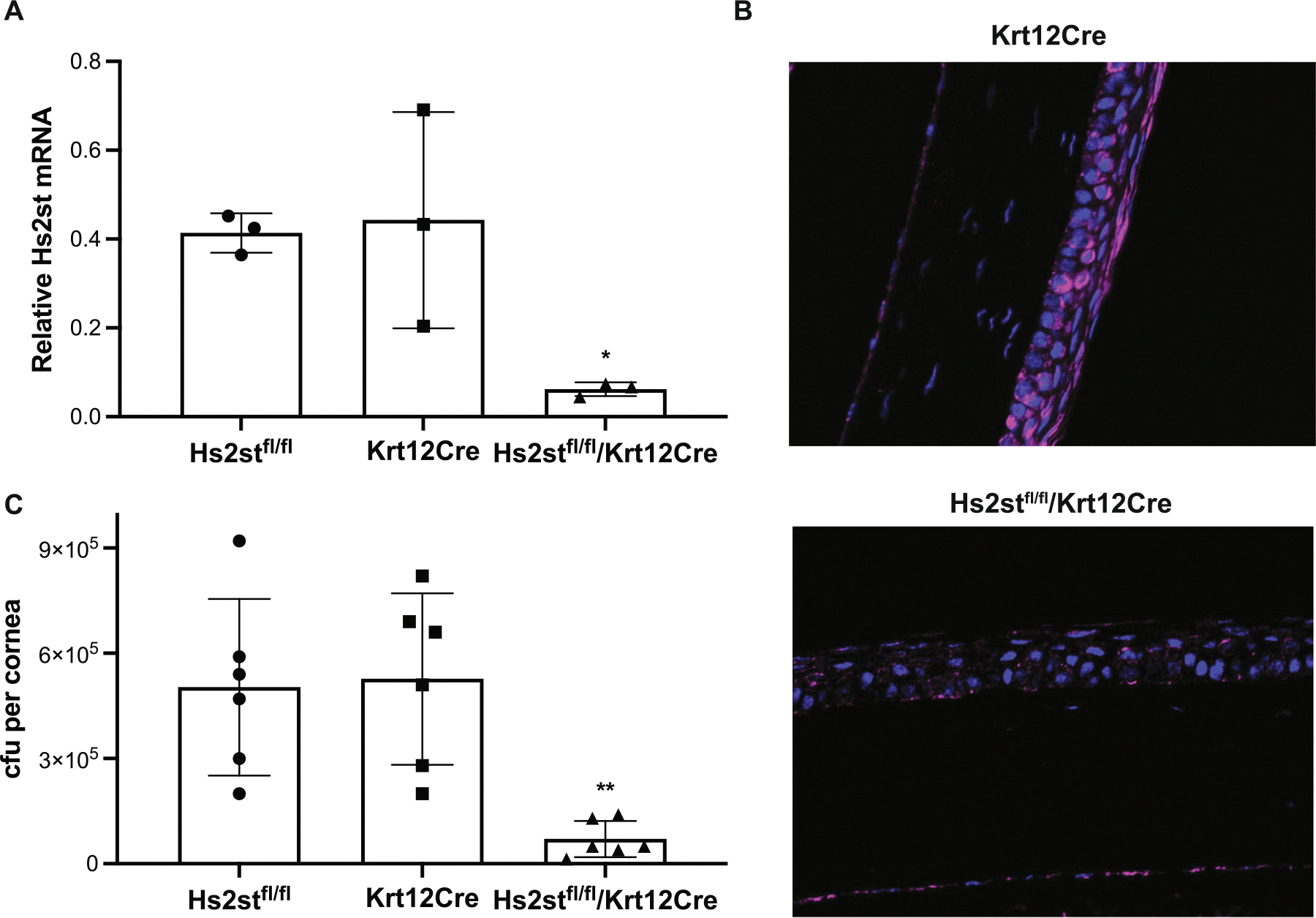
Conditional *Hs2st* deletion in corneal epithelial cells significantly inhibits *Staphylococcus aureus* corneal infection. (A) Relative Hs2st mRNA levels in the corneal epithelium of Hs2st^fl/fl^, Krt12Cre, and Hs2st^fl/fl^/Krt12Cre mice obtained by overnight dispase digestion of isolated corneas (*n* = 3, *p < 0.05). (B) Corneal sections of Krt12Cre and Hs2st^fl/fl^/Krt12Cre mice were immunostained for Hs2st with affinity-purified rabbit anti-mouse Hs2st and Alexa 647-conjugated goat anti-rabbit antibodies and counterstained with DAPI (original magnification, ×200). (C) Injured corneas of Hs2st^fl/fl^, Krt12Cre, and Hs2st^fl/fl^/Krt12Cre mice were infected with 3 × 10^7^ cfu of *S. aureus* USA300 in 5 μL PBS, and the bacterial burden was determined at 7 h pi (*n* = 6, **p < 0.01).

Corneal HS from mice and rabbits contains 2-*O*-sulfated uronic acid residues [50], suggesting that the heparan compounds described above mimic 2-*O*-sulfated HS present in the cornea. To establish that corneal 2-*O*-sulfated HS indeed promotes *S. aureus* pathogenesis in vivo, we tested the response of conditional knockout mice lacking *Hs2st* in keratin 12 (Krt12)-positive cells and, hence, devoid of 2-*O*-sulfated HS in the corneal epithelium. Floxed Hs2st (Hs2st^fl/fl^) [51] and Krt12Cre [52] mice were crossed to generate Hs2st^fl/fl^Krt12Cre^+/−^ mice. Hs2st mRNA levels were decreased by over 85% in the isolated Hs2st^fl/fl^Krt12Cre^+/−^ corneal epithelium compared to those of Hs2st^fl/fl^ and Krt12Cre^+/−^ mice (Figure 7A). Consistent with these data, HS2ST protein was markedly decreased in the corneal epithelium, but not endothelium, of Hs2st^fl/fl^Krt12Cre^+/−^ mice compared to that of Krt12Cre mice by immunostaining (Figure 7B). The conditional deletion of Hs2st did not affect the expression of Ndst1 (Figure S5A) and syndecan-1 (Figure S5B) as they were similar in the corneal epithelium of Hs2st^fl/fl^, Krt12Cre, and Hs2st^fl/fl^Krt12Cre^+/−^ mice. Importantly, when injured corneas of Hs2st^fl/fl^, Krt12Cre, and Hs2st^fl/fl^Krt12Cre^+/−^ mice were infected with 3 × 10^7^ cfu of *S. aureus* USA300, we found that the corneal bacterial titer is significantly reduced by over 86% in Hs2st^fl/fl^Krt12Cre^+/−^ mice compared to Hs2st^fl/fl^ and Krt12Cre^+/−^ mice (Figure 7C). Altogether, these data indicate that corneal epithelial HS binds and inhibits cathelicidins in a 2-*O*-sulfate-dependent manner and promotes *S. aureus* corneal infection.

## 3 | Discussion

We demonstrate for the first time that deletion of CRAMP leads to increased susceptibility in a mouse model of *S. aureus* corneal infection. CRAMP expression was induced by 4 h pi, and the effects of CRAMP deletion were not *S. aureus* strain-dependent, suggesting that CRAMP is mobilized as a major host defense factor against *S. aureus* corneal infection. The rapid induction of CRAMP upon infection and the fact that neutrophils are essential in the clearance of bacteria in murine *S. aureus* keratitis [53, 54] suggest that both epithelial cells and recruited neutrophils produce CRAMP. Neutrophil cathelicidins are stored in specific granules and are recruited to phagolysosomes for the phagocytic killing of *S. aureus* [55, 56]. Because soluble CRAMP can also efficiently kill *S. aureus* [39], these observations suggest that CRAMP kills *S. aureus* in both extracellular and intracellular compartments.

We also provide genetic and biochemical evidence that corneal epithelial HS promotes *S. aureus* corneal pathogenesis by inhibiting CRAMP in a 2-*O*-sulfate-dependent manner. We show that heparan compounds that contain 2-*O*-sulfated motifs potently and selectively inhibit *S. aureus* killing by CRAMP. Conditional deletion of Hs2st, the enzyme that catalyzes 2-*O*-sulfation of uronic acids in HS and HP, in the corneal epithelium significantly lowers the susceptibility to *S. aureus* corneal infection in mice. Furthermore, exogenous HS and HP inhibit the bacterial killing activity of cathelicidins produced by four different species, including human LL-37, suggesting that *S. aureus* may exploit epithelial HS to promote its pathogenesis in human keratitis and potentially other *S. aureu*s diseases. The major source of HS is likely syndecan-1, because epithelial cells express large amounts of syndecan-1 on their cell surface [57] and *S. aureus* activates syndecan-1 shedding via α-toxin [26], a major virulence factor of *S. aureus* keratitis [58]. However, several other HSPGs are also expressed in the cornea, including perlecan [59], syndecan-4 [60], and most likely several glypicans that are expressed on the cell surface as a glycosylpho-sphatidylinositol (GPI)-anchored HSPG. These HSPGs may also bind and inhibit cathelicidins, thereby promoting pathogenesis. In this regard, it is interesting to note that *S. aureus* expresses a phosphatidylinositol-specific phospholipase C (PI-PLC) [61], a virulence factor that should shed glypican ectodomains by cleaving the GPI linkage.

Many GAG–protein interactions depend on the overall net charge of the GAG rather than on specific sulfate modifications [37]. However, there are examples where a specific modification is critical. For example, several studies have shown that HS interactions with FGFs and Wnt are dependent on the presence of 2-*O*-sulfated uronic acids [62–65]. Uptake of lipoproteins by hepatocytes has also been shown to be dependent on 2-*O*-sulfated HS motifs in both cell-based assays and in mice [51, 66]. HS chains on syndecan-1 have also been suggested to interact with fibronectin in a 2-*O*-sulfate-dependent manner [67]. Our results indicate that HS-cathelicidin interactions also depend on 2-*O*-sulfates. Precisely how 2-*O*-sulfates regulate HS binding to cathelicidins is not known. Cathelicidins self-assemble into densely packed amphipathic α-helices, and the alternating hydrophobic and positively charged amino acids are required for the binding and disruption of negatively charged bacterial membranes [44]. Consistent with this model, our studies showed that the secondary structure of CRAMP is crucial for binding to HP. Heat-treatment abolished CRAMP binding to HP, and 10-mer synthetic peptides spanning the mature CRAMP sequence did not bind to HP. These findings suggest that the amphipathic α-helical structure of cathelicidins orients cationic amino acids in a conformation that is complementary to the sulfate motifs of HS. 2-*O*-sulfates might be suitably spaced to bind their cationic amino acid partners in cathelicidins and strengthen the HS-cathelicidin interaction. The conformation of 2-*O*-sulfated uronic acids might also be important. The uronic acid of HP is ≥ 70% iduronic acid (IdoA), whereas HS contains a higher proportion of glucuronic acid (GlcA) than IdoA, and H is strictly GlcA [68, 69]. The conformation of IdoA can be either a ^1^C4 chair, ^2^S0 skew boat, or ^4^C1 chair form, whereas GlcA prefers a ^4^C1 chair conformation [70, 71], suggesting that 2-*O*-sulfates on uronic acids in the ^4^C1 chair conformation might be important. However, more rigorous structural studies are needed to address these possibilities.

Currently, we do not know whether *S. aureus* actively exploits host HS to inhibit cathelicidins for its pathogenesis or if the host actively mobilizes HS to defend against potentially harmful effects of cathelicidins. Inhibition of LL-37 by GAGs has also been proposed to promote pathogenesis in cystic fibrosis, sepsis, and wound infection in humans [29–31]. Expressing a factor that inhibits host defense is, in principle, detrimental in infections, implying that HS inhibition of cathelicidins must serve one or more beneficial purposes. In fact, cathelicidins have a plethora of biological activities [6], including activation, differentiation, and recruitment of leukocytes [72–75], activation of complement and mast cell degranulation [3], stimulation of angiogenesis [76], and induction of cytokines and chemokines [3, 77, 78]. LL-37 can also bind to nucleic acids and facilitate TLR interactions with DNA and RNA [79, 80]. Furthermore, LL-37 can stimulate many signaling pathways, including p38, ERK, and JNK MAP kinases, NF-κB, PI3K-Akt, Src family kinases, Wnt-β-catenin, and JAK-STAT, among others [2, 3]. Excessive signaling can disrupt cellular homeostasis, leading to tissue damage and tumor formation. In addition, LL-37 has been shown to cause cell apoptosis and necrosis [81, 82] and contribute to the pathogenesis of diseases, such as psoriasis and rosacea [77, 83, 84]. These data indicate that cathelicidins are pro-inflammatory and implicated in a variety of inflammatory and tissue injury disorders that are not associated with infection. While these observations raise questions about the primary biological role of cathelicidins, they nonetheless suggest that one of the key reasons for the endogenous inhibition of cathelicidin peptides by HS is to keep their activities in check and prevent inflammatory tissue damage. In fact, a small clinical study showed that topical skin administration of a low molecular weight HS analog significantly improved clinical signs of rosacea, likely by inhibiting LL-37-induced IL-8 chemokine release [85]. We propose that it may be worthwhile to investigate the protective effects of defined 2-*O*-sulfated heparan compounds in inflammatory disorders involving cathelicidins.

## 4 | Experimental Procedures

### 4.1 | Materials

CRAMP, CAP-18, LL-37, and SMAP-29 peptides were purchased from Anaspec (Fremont, CA). Porcine mucosal HS, HP, NDS-HP, 2ODS-HP, and 6ODS-HP were from Neoparin (Alameda, CA). CS-A, DS, goat serum, rat serum, and Affi-Gel HP beads were obtained from Sigma-Aldrich (Waltham, MA). H was purified from *E. coli* K5 and chemoenzymatically *N*-sulfated or *N*- and 2-*O*-sulfated as described [67]. Lightning-Link Rapid FluoProbes647H labeling kit was from Novus Biologicals (Centennial, CO). BacLight Green bacterial stain was obtained from Thermo Fisher (Waltham, MA). Human anti-PNAG monoclonal antibodies (F598) were a generous gift from Dr. Gerald Pier (Harvard Medical School, Boston, MA) [40]. Rabbit anti-mouse HS2ST polyclonal antisera were generated in-house against a synthetic peptide with a sequence of C^190^RKQGDKKTFDECVAEGG^206^, with an N-terminal Cys added for coupling purposes. Anti-mouse HS2ST antibodies were purified by protein A and HS2ST peptide affinity chromatography. Alexa 647-conjugated goat anti-rabbit and Alexa 647-conjugated rat anti-human antibodies were from Biolegend (San Diego, CA). Oligonucleotide PCR primers were purchased from Integrated DNA Technologies (Coralville, IA). All other materials were purchased from Sigma, Thermo Fisher Scientific, or Avantor Sciences (Radnor, PA).

### 4.2 | Mice

Wt and *Camp*^−/−^ mice on the BL/6J background were purchased from Jackson Laboratory (Bar Harbor, ME). Krt12Cre mice [52] and floxed Hs2st (Hs2st^fl/fl^) [51] mice were crossed to generate Hs2st^fl/fl^Krt12Cre^+/−^ mice. All mice are healthy with normal growth, reproduction, tissue morphology, and CBC and serum chemistry parameters. Both female and male mice were used at an age of 6–10 weeks, except for the Hs2st^fl/fl^Krt12Cre^+/−^ mice and their controls which were used at 12–14 weeks. Mice were maintained in microisolator cages under specific pathogen-free conditions in a 12 h light/dark cycle and fed a basal rodent chow ad libitum. All animal experiments were approved by the Institutional Biosafety Committee and Institutional Animal Care and Use Committee of Boston Children’s Hospital and complied with federal guidelines for research with experimental animals.

### 4.3 | Mouse Model of *S. aureus* Corneal Infection

*S. aureus* strains USA300 [86], 8325–4 [26], and P1 [87] were from our culture collection. *S. aureus* was grown to late log phase in tryptic soy broth (TSB). The bacterial concentration was approximated by turbidity measurement at OD600 and adjusted. The exact bacterial dose was determined by plating serial dilutions of the inoculum immediately after infection and counting colonies the following day. Mouse corneas were injured by a single vertical scratch with a 29G needle in one of the corneas of each anesthetized mouse without penetrating beyond the superficial stroma. Injured corneas were infected topically with the indicated dose of *S. aureus* without or with test reagents in 5 μL PBS, and eyes were enucleated at 2–10 h pi. The bacterial burden in isolated corneas was determined by homogenizing corneas in TSB containing 0.1% (v/v) Triton X-100, plating out serial dilutions of corneal homogenates onto tryptic soy agar (TSA) plates, and counting colonies the following day.

### 4.4 | Quantitative Real-Time PCR

Corneas were dissected from enucleated eyes under a stereomicroscope, and the corneal epithelium was isolated after an overnight incubation with 10 mg/mL dispase II in DMEM/F12 at 4°C. RNA was isolated using the RNeasy Plus minikit. RNA concentration was determined with NanoDrop Lite (Thermo Fisher). qRT-PCRs were performed with 2 ng RNA using the following primers. Mouse Sdc1: AGGATGGAACTGCCAATCAG (forward), ATCCGGTACAGCATGAAAGC (reverse); mouse Ndst1: CCCAGTGGCCCTAAAGTACA (forward), GTCCATGACTTTGGCAGGTT (reverse); mouse Hs2st: CGACTGTGCTCCAGAGAAGC (forward), GCGATAGTCTGCTTGGTGGG (reverse); mouse Cramp: ATCAGCTGTAACGAGCCTGG (forward), AGGCCTACTACTCTGGCTGA (reverse); and mouse β-actin: ACCGTGAAAAGATGACCCAG (forward), GTACGACCAGAGGCATACAG (reverse), and a Verso 1-step RT-qPCR kit on a CFX96 real-time system (Bio-Rad, Hercules, CA). Target gene expression was normalized to β-actin using quantification cycle (ΔCq) between the target genes and β-actin.

### 4.5 | Bacterial Killing by Cathelicidins

Late log phase *S. aureus* USA300 was washed with phosphate buffer (8.2 mM Na_2_HPO_4_, 1.8 mM KH_2_PO_4_, pH 7.4) and approximately 500 cfu were incubated with cathelicidins with or without excess GAGs for 2 h at 37°C as indicated. Viable bacteria were enumerated by plating onto TSA plates and counting colonies the following day. Results are shown as % bacterial killing, where 100% killing represents data derived from the group of bacteria incubated with cathelicidins only.

### 4.6 | Bacteria Live/Dead Staining

*S. aureus* USA300 was stained by BaclightGreen (Thermo Fisher) following the manufacturer’s instructions. Briefly, an overnight culture of *S. aureus* was resuspended in PBS and incubated with 100 μM BaclightGreen for 15 min at room temperature. Approximately 400 cfu of BaclightGreen-labeled *S. aureus* were incubated with 0.5 μM CRAMP in 20 μL of phosphate buffer for 30 min at room temperature. Bacteria were washed with phosphate buffer and incubated with 10 μg/mL propidium iodide for 15 min at 4°C. Bacteria were mounted on glass slides and images were captured with a fluorescence microscope (Zeiss Axiovert 40 CFL). Pictures were taken with the AxioCam MRm high-resolution camera.

### 4.7 | CRAMP Binding to Bacteria

Alexa 647 fluorophore was conjugated to CRAMP using the Lightning-Link Rapid FluoProbes647H labeling kit following the manufacturer’s instructions. *S. aureus* USA300 fixed in 2% paraformaldehyde for 20 min was resuspended in 20 μL of phosphate buffer and incubated with 0.5 μM of Alexa 647-labeled CRAMP for 30 min at room temperature without or with glycosaminoglycans. Unbound Alexa 647-labeled CRAMP was removed by washing with phosphate buffer, and Alexa 647-labeled CRAMP bound to bacteria was assessed by flow cytometry.

### 4.8 | CRAMP Binding to HP

Affi-Gel HP beads were washed and resuspended in PBS. Affi-Gel HP beads (20 μL) were incubated with 0.5 μM Alexa 647-labeled CRAMP without or with 20 μg/mL HP, chemically desulfated HP, or chemoenzymatically sulfated H for 1 h at room temperature. Affi-Gel HP beads were washed with PBS, and the Alexa 647-labeled CRAMP bound to them was assessed by flow cytometry.

### 4.9 | ITC

ITC of CRAMP binding to HP and modified HP was carried out at 25.0°C using a MicroCal iTC 200 titration calorimetry system (Malvern, UK). A stock solution of CRAMP was diluted to 12.5 μM in 50 mM HEPS, pH 7.4, 150 mM NaCl, and approximately 300 μL of this solution was loaded into the ITC cell and titrated with 25 μM of HP or 2ODS-HP in the same buffer. Briefly, titration injection of HP or 2ODS-HP consisted of a first injection of 0.4 μL followed by 18 consecutive injections of 2 μL of 0.8 s duration and with a 2.5 min interval between injections. The resulting data were fitted into a single set of identical sites model using the MicroCal ORIGIN 70 software. The enthalpy change of binding (ΔH), the binding affinity, and the binding stoichiometry were determined.

### 4.10 | Histopathology

Enucleated eyes were fixed in 4% paraformaldehyde/PBS for 4 h at room temperature, paraffin-embedded, sectioned horizontally (5 μm), and mounted onto slides. Slide samples were deparaffinized, hydrated in xylene, 100% to 50% ethanol gradient, and PBS, and immunostained. For antigen retrieval, samples were microwaved twice in defrost mode for 6 min in 50 mM Tris (pH 8.8) with 1 mM EDTA and quenched with 100 mM NH4Cl. Samples were blocked with 10% (v/v) nonimmune serum of secondary antibodies in PBS and immunostained with rabbit anti-Hs2st polyclonal antibody (10 μg/mL) and Alexa 647-conjugated goat anti-rabbit antibodies or human anti-PNAG monoclonal antibody (17 μg/mL) and Alexa 647-conjugated goat anti-human antibodies. Images were captured with the Zeiss Axiovert 40 CFL microscope, and pictures were taken with the AxioCam MRm high-resolution camera. Adobe Photoshop 26.4 was used to process the acquired images.

### 4.11 | Data Analyses

Data are expressed as mean ± SD. Statistical significance between experimental and control groups was analyzed by unpaired Welch’s *t*-test and between multiple groups by ANOVA followed by Dunnett’s post hoc test using GraphPad Prism (version 10.4.1e). *p* ≤ 0.05 was determined to be significant.

## Supplementary Material

Fig.S2

Fig.S1

Fig.S3

Fig.S4

Fig.S5

Supporting Information

Additional supporting information can be found online in the Supporting Information section.

**Figure S1.** A) CRAMP expression was measured in uninfected Wt corneas or Wt corneas isolated at 4 h post-*S. aureus* USA300 infection. Briefly, eyes were enucleated, corneas were dissected, and isolated corneas were incubated with 10 mg/ml dispase II overnight at 4°C to peel off the corneal epithelium. Total RNA was purified from the corneal epithelium, and 1 ng of total RNA was analyzed by qRT-PCR for CRAMP or β-actin expression (*n* = 3, **p < 0.01). B) Dose response of *S. aureus* killing by cathelicidins was assessed by incubating 500 cfu of *S. aureus* USA300 with 0, 2, 20, 250, 500, or 1000 nM of CRAMP, LL-37, CAP-18, or SMAP-29 for 2 h at 37°C. Bacterial killing was measured by plating serial dilutions onto TSA plates and counting colonies the following day. Results are shown as % bacterial survival (*n* = 4). **Figure S2.** A) Dose-dependence of HS inhibition of bacterial killing by CRAMP was assessed by incubating 500 cfu of USA300 with 0.5 μM CRAMP with 0, 10, 100, 500, or 1000 ng/ml HS for 2 h at 37°C. Bacterial killing (%) was enumerated by plating serial dilutions onto TSA plates and counting colonies the following day (*n* = 5). B) Effects of pre-incubating *S. aureus* with glycosaminoglycans on bacterial killing by cathelicidins were examined by incubating 500 cfu of USA300 without or with 5 μg/ml HS, HP, DS, or CS for 30 min at 37°C. Bacteria were washed three times with buffer, incubated with 0.5 μM CRAMP for 2 h at 37°C, and % bacterial killing was determined by plating serial dilutions of samples (*n* = 4). **Figure S3.** *S. aureus* USA300 stained with BaclightGreen was incubated with 0.5 μM CRAMP in the absence or presence of 5 μg/ml HP, NDS-HP, 2ODS-HP, 6ODS-HP, H, NS-H, or NS2OS-H for 30 min at room temperature. Bacteria were washed and incubated with 10 μg/ml propidium iodide for 15 min on ice. Images were captured with a fluorescence microscope (Zeiss Axiovert 40 CFL). Quantification of % dead bacteria (out of total bacteria in field) is shown at the bottom. **Figure S4.** Alexa 647-conjugated CRAMP (0.5 μM) was incubated with fixed *S. aureus* USA300 without or with 5 μg/ml of the indicated glycosaminoglycans and chemically-desulfated HP compounds for 1 h at room temperature. *S. aureus*-associated CRAMP fluorescence was analyzed by flow cytometry. Representative FACS density plots are shown where heparan compounds containing 2-*O*-sulfated motifs decreased Alexa CRAMP binding by over 79% compared to the control (No GAG), except for NDS-HP where the reduction was approximately 65%. **Fig. S5.** Relative mRNA levels of A) Ndst1 and B) Sdc1 in the corneal epithelium of Hs2st^fl/fl^, Krt12Cre, and Hs2st^fl/fl^/Krt12Cre mice (*n* = 3).

## Data Availability

All data are contained within the manuscript.
