# Supplementary figures and images for: Epithelial Heparan Sulfate Promotes Staphylococcus aureus Corneal Infection by Inhibiting Cathelicidins
Source: Proteoglycan Res. Author manuscript; Available in PMC 2026 Jan 16. (PMC12806164; doi:10.1002/pgr2.70041)

A

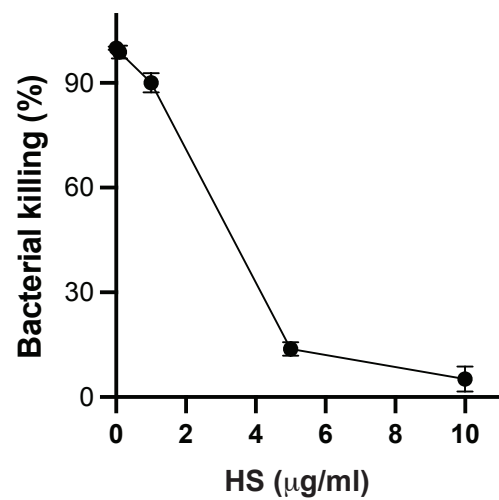

B

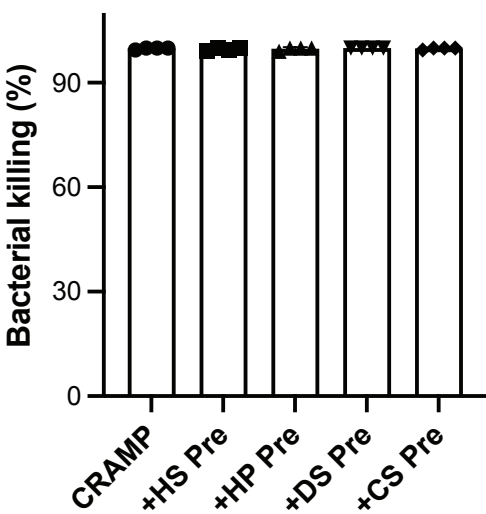

Supplement: Fig.S2 [file NIHMS2121318-supplement-Fig_S2.pdf]

**A**

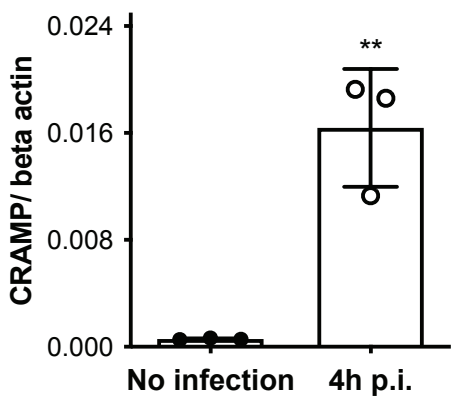

**B**

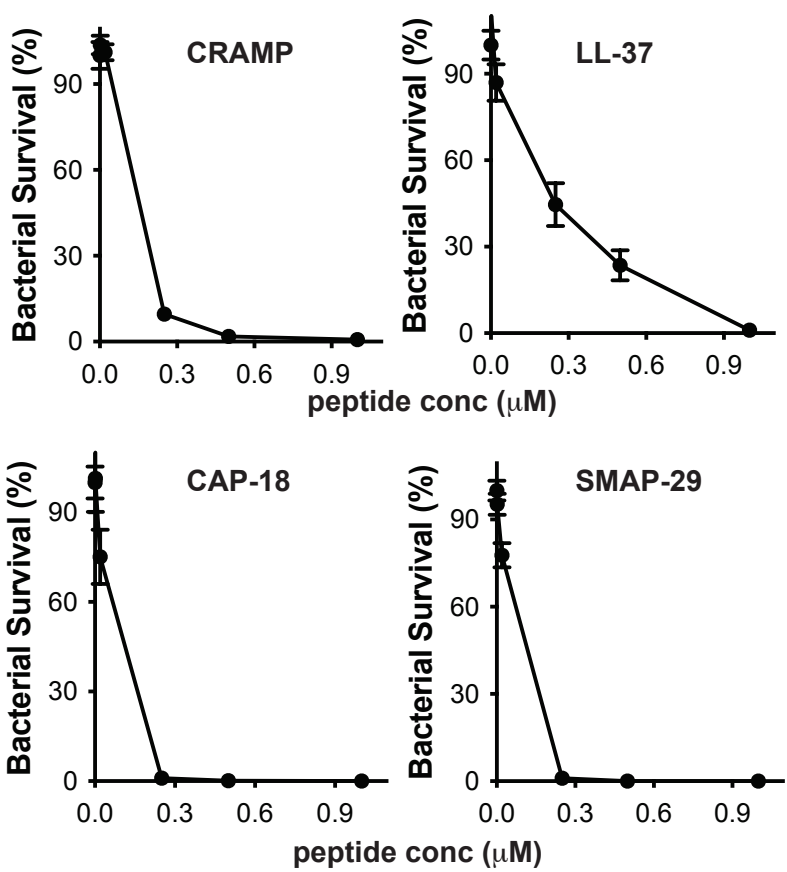

Supplement: Fig.S1 [file NIHMS2121318-supplement-Fig_S1.pdf]

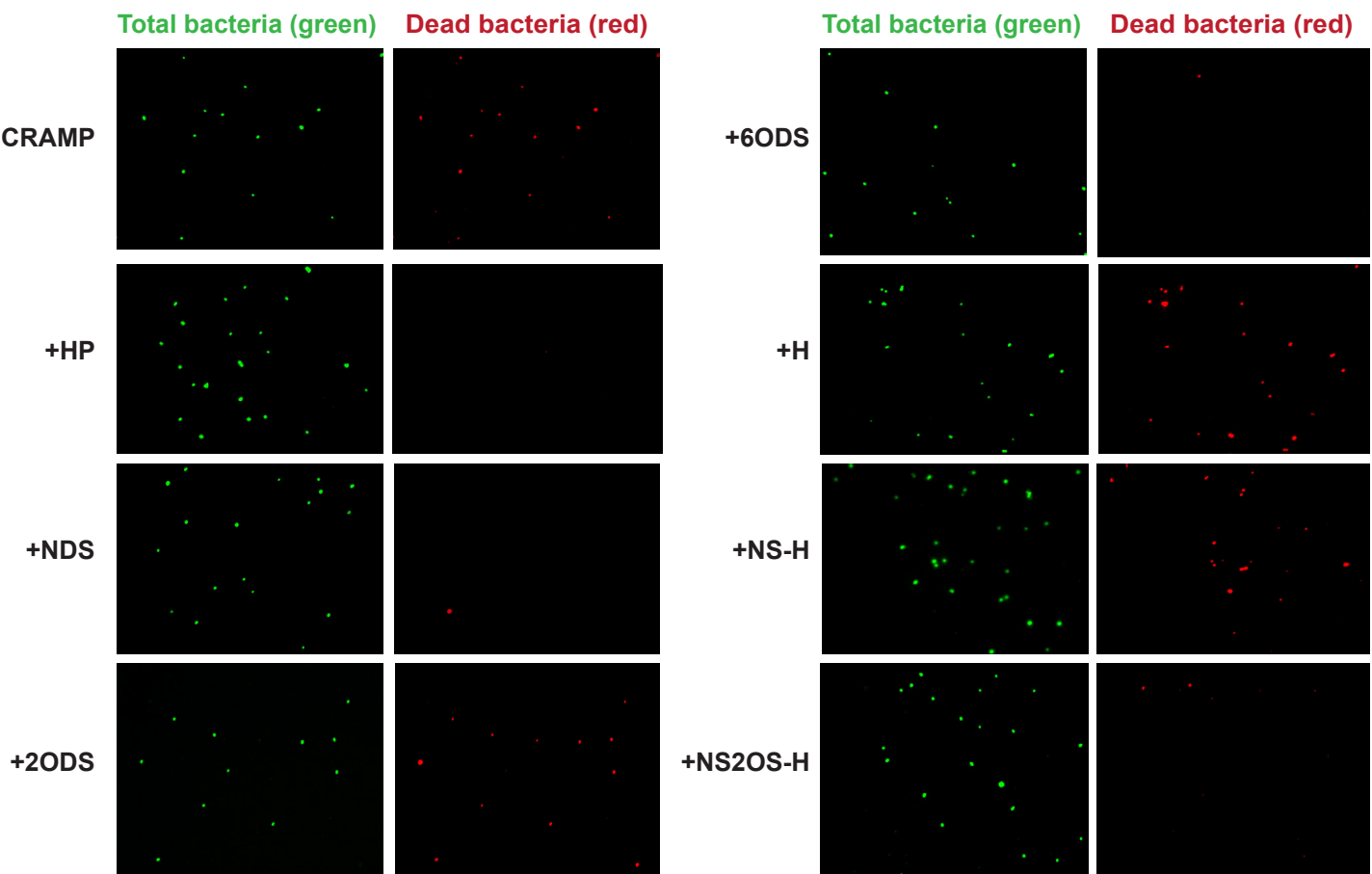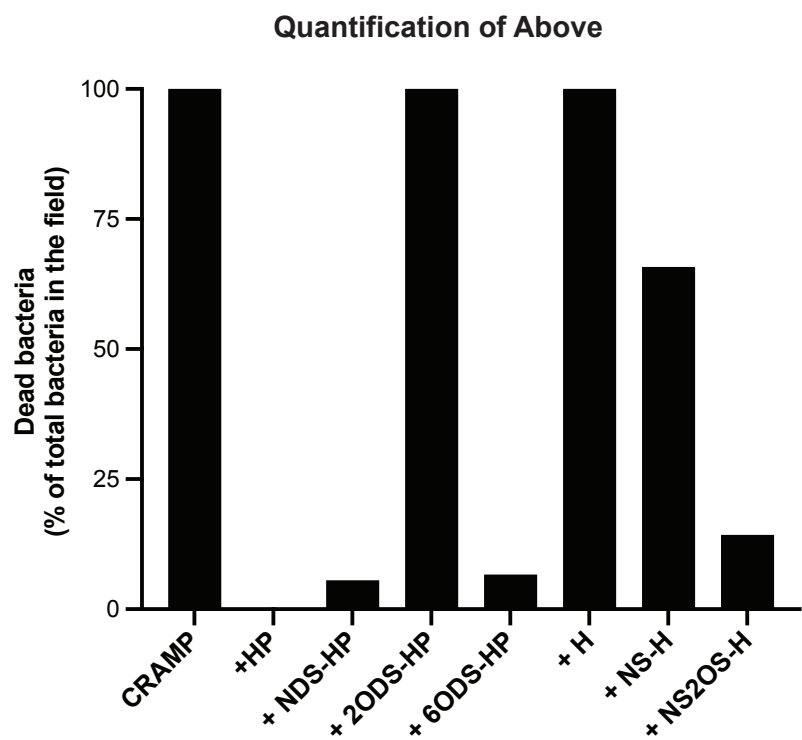

Supplement: Fig.S3 [file NIHMS2121318-supplement-Fig_S3.pdf]

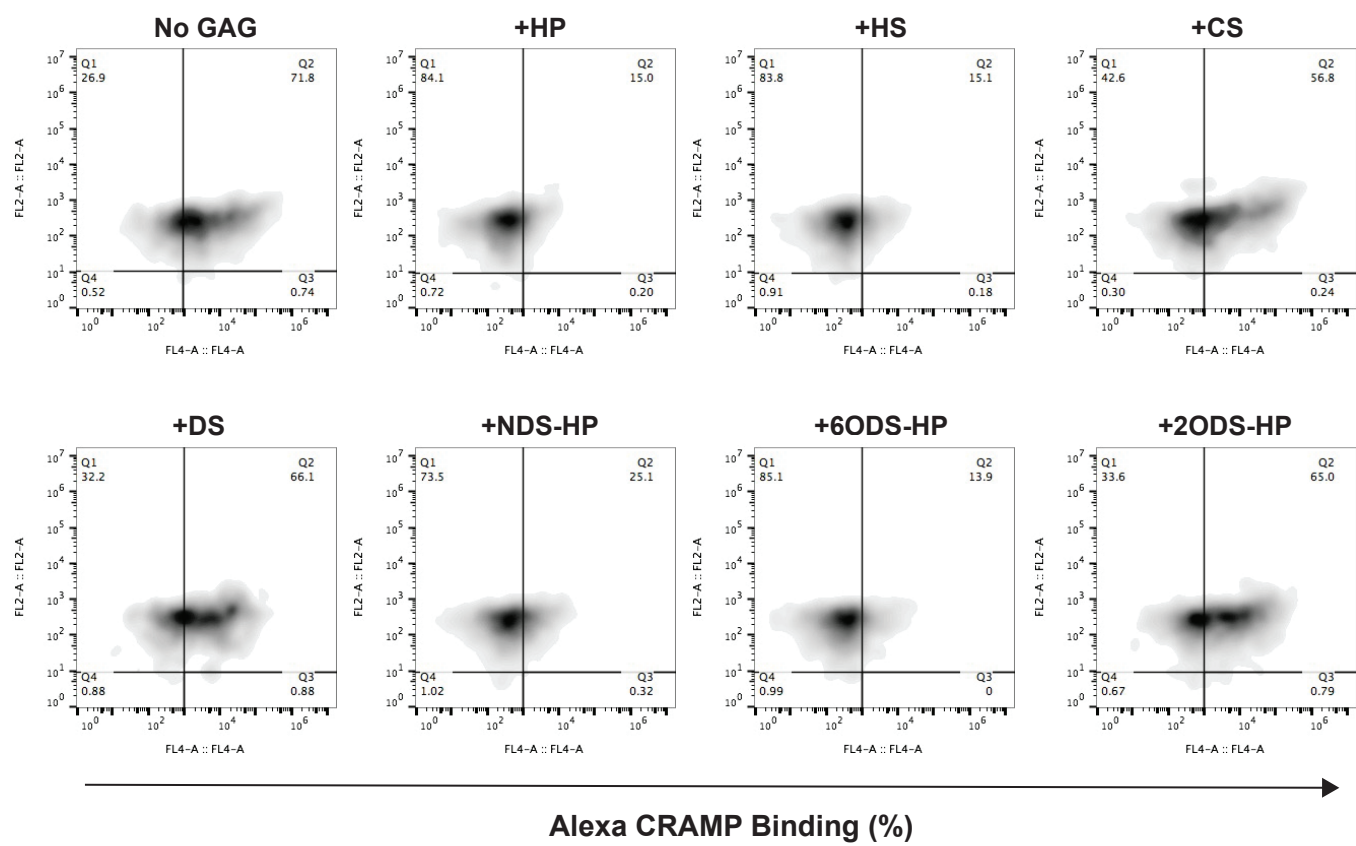

Supplement: Fig.S4 [file NIHMS2121318-supplement-Fig_S4.pdf]

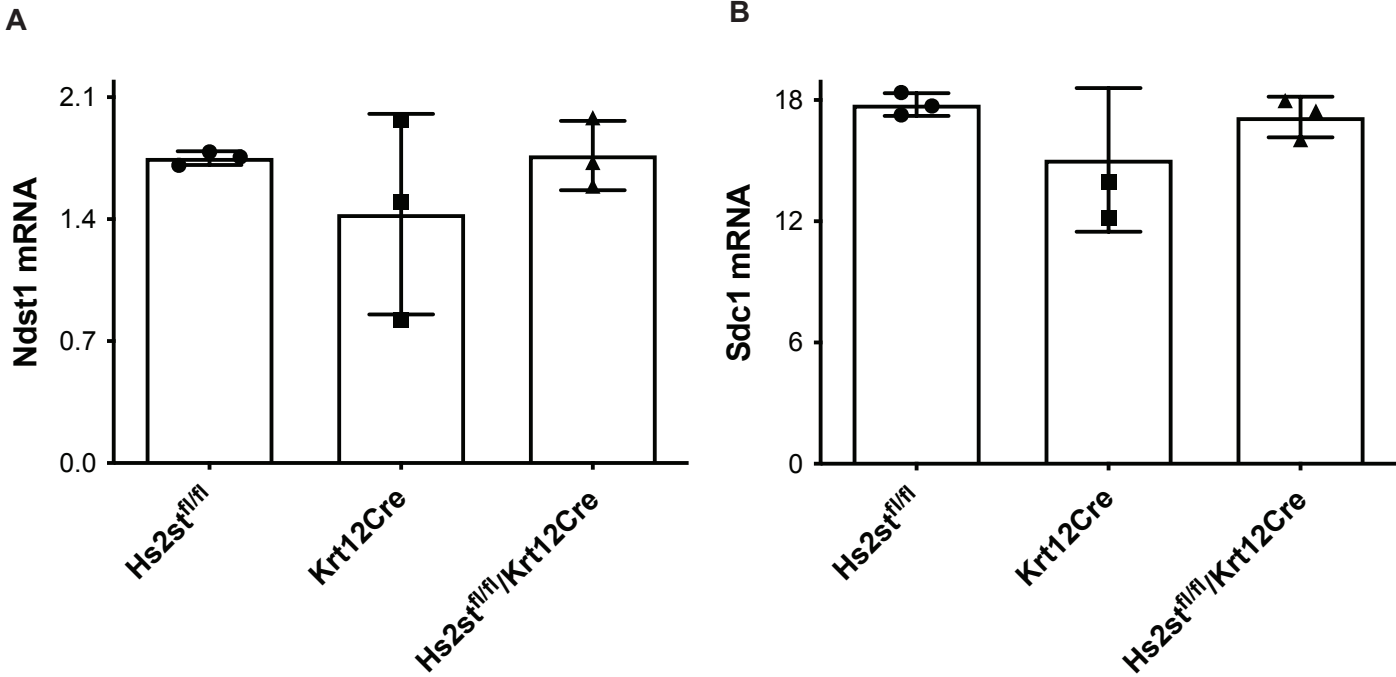

Supplement: Fig.S5 [file NIHMS2121318-supplement-Fig_S5.pdf]
